# Pre-eclampsia as Underlying Cause for Perinatal Deaths: Time for Action

**DOI:** 10.9745/GHSP-D-15-00350

**Published:** 2015-12-15

**Authors:** Stephen Hodgins

**Affiliations:** ^a^​ Global Health: Science and Practice, Deputy Editor-in-Chief, Washington, DC, USA.

## Abstract

Pre-eclampsia is a major underlying cause of late fetal and early neonatal death, accounting for somewhere between 1 in 10 and 1 in 4 perinatal deaths; it warrants greater efforts from the maternal-newborn community.

The Global Maternal Newborn Health Conference held in Mexico City in October 2015 marks an important watershed in global efforts to reduce the burden of preventable maternal and newborn deaths, bringing together—as it did—what have been two fairly distinct technical communities (maternal and newborn) to tackle their shared challenges in a post-Millennium Development Goal era. With this broadening focus embracing both mother and fetus/newborn, it is an appropriate time to reflect on where we may be allowing things to fall through the cracks and how—with a more seamless effort—we can do better.

A first important observation is that although there has been growing attention in global health to maternal and newborn health and each of these areas has an active constituency, stillbirth remains relatively neglected. Yet it represents one of the starkest examples of global inequity. Risk of stillbirth is 10 times higher in South Asia and sub-Saharan Africa than it is in high-income countries.^1^^,^^2^ To focus our attention on where there is the greatest burden of preventable mortality, it can be helpful to reframe our view of mortality in a way that captures deaths late in pregnancy, during labor, and in the first hours and days after birth, recalling that globally it is estimated 73% of newborn deaths occur within 7 days of birth.^3^ Perinatal mortality comprises fetal deaths late in pregnancy (≥28 weeks gestation) and during labor, and newborn deaths through the first week after birth.

## ROLE OF PRE-ECLAMPSIA IN MATERNAL HEALTH

Pre-eclampsia and the life-threatening condition of eclampsia (seizures associated with this disorder) constitute an important contributor to the burden of bad maternal-newborn outcomes. Eclampsia/pre-eclampsia accounts globally for about *1 in 7 maternal deaths*,^4^ with most (in high-mortality settings) resulting from eclampsia. In sub-Saharan Africa, 1 of every 1,500 pregnancies ends in a maternal death attributable to eclampsia/pre-eclampsia; in South Asia the proportion is about 1 in 3,000 (calculated from Kassebaum^5^). The importance of the problem *has* been recognized within the maternal health community, and this is reflected in the emphasis it has placed on use of magnesium sulfate for care of women with eclampsia and severe pre-eclampsia, for example, as one of the Emergency Obstetrical Care “signal functions.”^6^

Eclampsia/pre-eclampsia accounts globally for about 1 in 7 maternal deaths.

## KEY ROLE OF PRE-ECLAMPSIA IN PERINATAL MORTALITY

Although not very evident in global newborn strategy documents, *eclampsia/pre-eclampsia makes a similarly important proportionate contribution to perinatal mortality*, and this translates into a far larger number of deaths. This effect is mediated through compromised fetal nutrition and oxygenation resulting from utero-placental vascular insufficiency (Box). According to data from a multicountry study conducted by the World Health Organization in Argentina, Egypt, India, Peru, South Africa, and Viet Nam, which included just under 8,000 pregnancies enrolled during antenatal care,^8^ eclampsia/pre-eclampsia was the primary obstetrical cause for 1 of 4 perinatal deaths, with similar proportions affected for stillbirths and early newborn deaths. In this study, data were captured until discharge or day 7 postpartum, whichever happened first. Stillbirths were included if they weighed ≥1,000 g or, if weight was unavailable, if they had reached 28 weeks gestation.

**BOX. Effect of Pre-eclampsia on Fetal Growth**

Pre-eclampsia is characterized by poor utero-placental circulation secondary to inadequate remodeling of the spiral arteries that occurs between weeks 8 and 18. There may be many routes to pre-eclampsia with different contributions from the mother and the placenta. Two individuals are involved, mother and baby, each with different genetic make-ups. Placental vascular dysfunction, which can be particularly significant in early-onset disease, compromises nutrition and oxygenation of the fetus and is associated with fetal growth restriction.^7^

A more recently published and far larger hospital-based study (with more than 300,000 pregnancies) was conducted in 29 low- and middle-income countries.^9^^,^^10^ The study explored the relationship between severe, life-threatening maternal complications and perinatal deaths. It found that such complications were the underlying obstetrical cause of 23% of macerated late fetal deaths, 28% of fresh late fetal deaths, and 21% of early neonatal deaths.* The most important category of such obstetrical causes for perinatal deaths was hypertensive disorders, with life-threatening eclampsia and pre-eclampsia underlying 7.5% of macerated late fetal deaths, 9% of fresh late fetal deaths, and 10% of early neonatal deaths.

These two studies differed in the epidemiology of their study populations and were not measuring antecedent cause in the same way (primary obstetrical cause vs. life-threatening maternal complication), but they give a similar picture of a very important contribution of eclampsia and severe pre-eclampsia to perinatal mortality, ranging from about 1 in 10 perinatal deaths up to 1 in 4 (again, depending on local epidemiology and the methodologies used). This puts the impact of pre-eclampsia into the same range as 3 of the 4 most important *proximate* causes of early newborn deaths (intrapartum complications, 27%; congenital anomalies, 10%; and sepsis, 8%, according to Lawn^3^).^†^ Rates of perinatal deaths were similar in these two studies (12.5 late fetal and 9 early neonatal deaths per 1,000 births in Ngoc^8^; 18 late fetal deaths and 8 early neonatal deaths per 1,000 births in Vogel^9^) (Figure).

As an underlying cause, pre-eclampsia/eclampsia is similar in its contribution to perinatal mortality as the major immediate causes of intrapartum complications, congenital anomalies, and sepsis.

**FIGURE.**
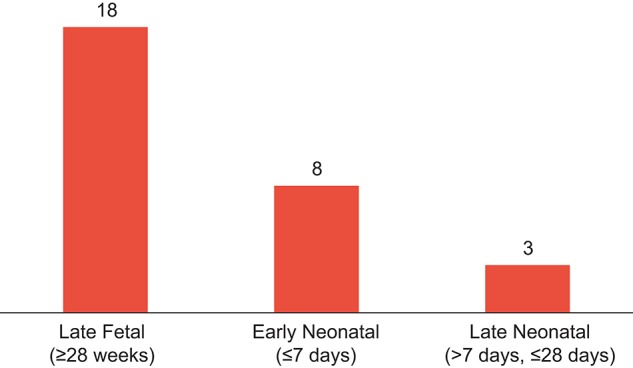
Perinatal Deaths per 1,000 Births Attributable to Eclampsia and Pre-eclampsia Source: Vogel et al. 2014^9^ and corrigendum by Vogel et al. 2015^10^; for late neonatal, also Lawn 2014.^3^

## LARGELY UNRECOGNIZED. WHY?

Despite the important contribution of eclampsia/pre-eclampsia to perinatal and newborn deaths, it has—for the most part—been absent from strategies elaborated globally to try to reduce the burden of such deaths. One could speculate this has been due in part to those in the newborn community seeing this problem as falling in the maternal health domain. On the maternal health side—as noted above—there has been attention to trying to ensure that when women arrive in hospital in a life-threatening state of eclampsia or severe pre-eclampsia they are appropriately treated with magnesium sulfate (though there is no evidence this helps reduce perinatal deaths). However, serious programmatic attention has not extended much further. This represents an important missed opportunity to achieve better outcomes.

## WHAT’S NEEDED?

As Goldenberg has documented,^11^ in the United States in 1930, eclampsia-attributable maternal deaths were at levels similar to the current burden in high-mortality settings in Africa and South Asia. Over the following half-century (*before* introduction of magnesium sulfate), such mortality was reduced by about 99%, with over 90% of that decline due to reduced incidence of eclampsia achieved by early identification of pre-eclampsia (through routine antenatal care screening) and timely delivery. [Fn fn3]The Goldenberg review documents the same historical pattern across high-income countries. To date, unfortunately, this important lesson has not been widely applied in program efforts in low- and middle-income countries.

But if we want to take a big chunk out of the wedge of maternal, newborn, and stillbirth mortality attributable to eclampsia/pre-eclampsia, maternal and newborn communities need to join forces and ensure a more comprehensive effort that includes:^‡^

- Systematic early identification of pre-eclampsia (requiring frequent antenatal contacts, particularly over the last 2 months of pregnancy, which cannot be achieved with the current 4-visit schedule)
- Timely delivery (*before* the woman reaches a life-threatening state)
- Effective management of those cases that progress to a life-threatening state (including appropriate use of magnesium sulfate and antihypertensive drugs, as well as appropriate medical support)

Maternal and newborn health communities must join forces to prevent maternal, perinatal, and newborn mortality attributable to eclampsia/pre-eclampsia.

## Footnotes

* Late fetal deaths were defined as deaths to fetuses weighing ≥1,000 g at birth, or if birthweight was unknown, ≥28 weeks gestation. Macerated late fetal deaths were late fetal deaths with signs of maceration, or tissue degeneration, at birth; fresh late fetal deaths were late fetal deaths with no signs of maceration at birth; and early neonatal deaths were deaths of a live-born neonate by discharge or day 7 of life.

^†^​ Prematurity accounts for a larger proportion.

^‡^​ Note that antenatal calcium supplementation is a promising preventive intervention in populations with low calcium intake, possibly reducing risk of neonatal death by up to 30%^12^ and pre-eclampsia-specific maternal mortality by a similar proportion; however, dosage issues and implementation challenges remain.
